# 

**Published:** 1906-05

**Authors:** 


### BOOKS RECEIVED.

Ellis's Demonstrations of Anatomy. Being a guide to the knowledge of the human body by dissection. Twelfth edition. Revised and edited by Christopher Addison, M.D., BS. (Lond.), F. R. C. S. Lecturer on Anatomy, Charing Cross Hospital, Medical School; formerly Hunterian Professor, Royal College of Surgeons, England; Examiner in Anatomy, Royal College of Surgeons, England, etc. Illustrated by 306 engravings on wood, of which a large number are in colors. Octavo volume, 861 pages. New York: William Wood & Company. 1906. (Price, muslin binding, $3.50 net).

---

Diseases of the Nervous System resulting from Accident and Injury. By Pearce Bailey, A.M., M.D., Clinical Lecturer in Neurolcgy, Columbia University, New York; Consulting neurologist to Roosevelt, Saint Luke's, and Manhattan State Hospitals. Octavo, 628 pages. New York and London: D. Appleton and Company. 1906. (Price: Cloth, ???????)

---

Nursing in the Acute Infectious Fevers. By George P. Paul, M.D., Assistant Visiting Physician and Adjunct Radiographer to the Samaritan Hospital, Troy, N. Y. 12mo. volume of 200 pages, illustrated. Philadelphia and London: W. B. Saunders and Company. 1906. (Price, $1.50 net).

International Clinics. A Quarterly of Illlustrated Clinical Lectures and especially prepared articles on Treatment, Medicine, Surgery, Neurology, Pediatrics, Obstetrics, Gynecology, Orthopedics, Pathology, Dermatology, Ophthalmology, Otology, Rhinology, Laryngology, Hygiene and other topics of interest to students and practitioners. By leading members of the medical profession throughout the world. Edited by A. O. J. Kelly, A.M., M.D., Philadelphia. Volume I. Sixteenth series. 1906. Philadelphia and London: J. B. Lippincott Co. (Cloth, $2.00).

The Medical Directory of New York, New Jersej, and Connecticut. Published by the New York State Medical Association, under the direction of the committee of publication, Charles Ellery Dennison, chairman: E. Eliot Harris; Thomas F. Reilly; Henry Roth, and William Ridgely Stone. Volume VII. New York: 64 Madison Avenue. 1905. (Price, $2.50).

The International Medical Annual. A yearbook of Treatment and Practitioner's Index. Twenty-fourth year. Substantially bound in cloth and fully illustrated by plates in color and black and white. New York: E. B. Treat and Company. 1906. Octavo, 700 pages. (Price $3.00 net., post or express paid).

X-Rays in General Practice. By A. E. Walter, Captain I. M. S., Superintendent of the X-Ray institute of India. Duodecimo 174 pages. Illustrated. London: John Lane, The Bodley Head. New York: John Lane Company. 1906.

